# Access to health services during the Colombian armed conflict: a challenge for the population with disabilities in the department of Meta

**DOI:** 10.1186/s12913-023-09472-x

**Published:** 2023-06-13

**Authors:** Nicolás Rodríguez Caicedo, Sebastián León-Giraldo, Catalina González-Uribe, Oscar Bernal

**Affiliations:** 1 Alberto Lleras Camargo School of Government, Universidad de los Andes, Bogotá, 11711 Colombia; 2 School of Medicine, Universidad de los Andes, Bogotá, 11711 Colombia

**Keywords:** Disability, Colombia, Armed conflict, Access to health, Qualitative

## Abstract

**Background:**

The Colombian armed conflict has left millions of victims and has restricted access to different services provided by the government, especially for people with disabilities. This article studies the barriers faced by the victim population with disabilities when they want to access the health system in the department of Meta, Colombia, and offers a perspective from the experiences of people with disabilities who have been victims of the armed conflict in the country.

**Methods:**

To carry out this qualitative study, focus groups were conducted to capture the experiences and feelings of this population in the context of violence and high conflict.

**Results:**

The results show the barriers encountered by the victim population with disabilities, their families, and their caregivers when they want to access medical or health services.

**Conclusions:**

Many problems affect the population with disabilities and the victim population in Colombia today. The Colombian government has not been able to establish adequate policies to eliminate or even reduce access to services such as health, education, housing, and social protection.

**Supplementary Information:**

The online version contains supplementary material available at 10.1186/s12913-023-09472-x.

## Key message

• The population with disabilities who have been victims of the armed conflict in Colombia find more significant barriers to accessing health services than people without disabilities due to various situations of exclusion in the provision and accessibility of medical services.

• In the department of Meta, the population with disabilities who have been victims of the armed conflict manifest being vulnerable and lacking protection from the Colombian government.

• Colombia, to date, has not been able to fully implement national and international agreements that guarantee respect for the human rights of people with disabilities.

• It is essential that both national and local governments implement policies and actions so that the victim population with disabilities can access health, education, and social protection services.

**Supplementary Information:**

The online version contains supplementary material available at 10.1186/s12913-023-09472-x.

## Introduction

The Convention on the Rights of Persons with Disabilities [1] stipulates, in its article 25, the obligation on behalf of the States to provide quality, accessible, and free health services, adopting the appropriate measures to ensure access for persons with disabilities; these services should consider gender issues, including health-related rehabilitation. Unfortunately, in Colombia, it has been evidenced that, for the population with disabilities, in areas where the armed conflict has strongly affected the people, this fundamental right is limited [2], [3].

Colombia has approximately seven million people with disabilities, representing 7% of the country’s population [4]. About 80% of the population with disabilities lives in poverty, living in the lowest socioeconomic strata. Furthermore, 64% of this population receives no wage income [4]. Additionally, 40% of the said population, according to data from the Ministry of Health and Social Protection, is unschooled. Along with the problematic socioeconomic situation in which the disabled population finds itself, it has been shown that 13% of this population has also been a victim of the Colombian armed conflict [4].

Out of the 32 departments of the country, Meta has been characterized by its livestock, mining, and agricultural economy. This department, which is in the center of the country, very close to the capital Bogotá D.C, has stood out for the extraction of crude oil and gas [5]. However, this department also stands out as one of the most affected by the Colombian armed conflict. Between 1985 and 2016, the armed conflict left approximately 210,000 displaced people, 30,000 homicides, 13,000 cases of forced disappearance, 8,000 threats, and 2,500 terrorist acts in this department [6]. Additionally, 23,409 people with disabilities live in this department, of which 5,256 are victims of the Colombian armed conflict [4].

The report “Disability and Armed Conflict” [7], issued by The Geneva Academy of International Humanitarian Law and Human Rights, shows that the population with disabilities is often subjected to selective killings; they are used as human shields, women and girls are at a greater risk of being victims of sexual violence, and they are more likely to die or be seriously injured; all of the latter, due to inaccessible protection mechanisms and lack of timely medical assistance [7].

The use of landmines by armed groups in the Colombian conflict has been one of the major causes of physical, mental, psychological, or psychosocial disability [8–10]. In addition to these types of actions against the social and life projects of the population with disabilities in the country, according to the report “Disability: An absent story in the armed conflict” [11] presented by the Program of Action for Equality and Social Inclusion - PAIIS, people with disabilities in Colombia have been victims of sexual abuse, forced displacement, psychosocial impacts, and physical violence.

In addition to the effects of the Colombian conflict on the population with disabilities, it has been shown that in areas where the armed conflict is experienced, access to health systems and the provision of services is limited [12–14]. According to the report ¡Basta Ya! carried out by Centro de Memoria Histórica [15], armed groups directed their actions on controlling educational centers and health service providers. In addition, the same report exposes the misuse, by illegal groups, of elements and utensils used to develop health practices. For example, drugs and hospital beds were taken by armed groups and used for their own purposes, for their troops personal use or to carry their injured people or captured soldiers [15].

The population victim of the armed conflict has been shown to have more difficulty accessing health services in Colombia, specifically in the department of Meta [16].This situation is aggravated for the population with disabilities who have been victims of the armed conflict due to the double problem of exclusion they experience [17–19]. The different discriminatory and exclusionary actions are reflected in the inadequate provision of health services, poor practices by health specialists, barriers to access to facilities, and the lack of reasonable accommodations so that the population with disabilities can access health services [20–22].

This article aims to analyze how the population with disabilities in the department of Meta has seen their right to health and access to services provided affected due to the experiences and effects of the armed conflict. In addition, this article seeks to give visibility to the voices of the population with disabilities who have been victims of the conflict and expose the problems they experience due to the armed conflict. Finally, some recommendations will be presented regarding the inclusion of the population with disabilities in the department of Meta health system.

## Methodology

To develop this research, a descriptive qualitative study was carried out, which used focus groups to learn about the experiences and perceptions of the population victim of the armed conflict in different municipalities of the department of Meta. The focus group participants were selected using a snowball sampling technique. All were members of victim organizations known as “Mesas de Víctimas,“ indicating that they were organized and active in seeking justice and support for victims of armed conflict. Furthermore, as members of the “Mesas de Víctimas,“ which are spaces intended for discussion, dialogue, feedback, training, and monitoring of the provisions contained in Law 1448 of 2011 [23], some participants knew the situation of other victims in the region, including those who were disabled as a result of the conflict. Despite the fact that none of the participants themselves had a disability due to the armed conflict, several had close family members or loved ones who had become disabled due to the violence, and some were caregivers (such as mothers or sisters) to individuals who were disabled due to the conflict.

The Law 1448 of 2011, also known as the Victims and Land Restitution Law, is a landmark legislation in Colombia that seeks to provide reparations and assistance to victims of the country’s long-running internal armed conflict [23]. The law recognizes the rights of victims of violence, including those who have been displaced, murdered, or subjected to other forms of violence. It aims to provide them compensation, restitution, and support to rebuild their lives. The law establishes the creation of the National Victims’ Unit (Unidad de Víctimas), a government agency responsible for implementing the law and assisting victims. It also establishes mechanisms for land restitution, recognizing that many victims were forcibly displaced from their lands during the conflict. Overall, Law 1448 is an essential legal instrument in Colombia’s efforts to address the legacies of violence and promote a culture of peace and justice. Its provisions have been the subject of ongoing debates and controversies, but many advocates for victims’ rights consider it a crucial step toward reconciliation and healing.

It is important to clarify that although the research project that framed this paper did not specifically focus on disability, and there were no explicit questions related to disability, it was a common topic that arose during the focus groups. This paper is a result of an emergent analysis of the data that included important insights related to disability and its intersections with other forms of violence and victimization.

### Data collection

For data collection, focus groups were carried out. Using this research technique allows the participants, who attended voluntarily, to be able, through conversations and interactions, to generate knowledge, which is collected and organized for this research. The people who participated in these sessions were victims of the armed conflict and residents of the department of Meta.

The conversations carried out in the focus groups were organized and divided depending on the topics to be discussed, seeking to have groups of only men, only women, and mixed groups. Most of the people who responded to the call and participated in the focus groups were women. Nine focus groups were held in four municipalities of the department of Meta, summarized in Table 1. These focus groups were organized depending on the degree of intensity experienced in the armed conflict, a strategy suggested by the Resource Center for Conflict Analysis (CERAC).

**Table 1:** Focus groups by location, gender, and number of participants

Focus Group	Place	Men	Women
I	Villavicencio (capital city, strongly affected)	0	8
II		12	0
III		0	10
IV	Castilla La Nueva (without conflict)	0	6
V		4	4
VI	Granada (lightly affected)	0	6
VII		0	5
VIII		0	7
IX	Vistahermosa (strongly affected)	7	0
X		0	6
**TOTAL**		23	52

### Focus groups procedures

The focus groups were supported by local victims’ organizations in the department of Meta. This helped make it possible to locate all the participants in the different municipalities of the department. To participate in the focus groups, all participants had to sign an informed consent, which included an explanation of the goal of the focus group, its implications, and its scope. All participants were of legal age (18 years in Colombia).

The focus groups were organized in the month of September 2019. They lasted approximately one hour and were moderated by research assistants trained in the project. These sessions were conducted using a semi-structured guide, which allowed for any new issues or topics to be fully developed, considering opinions and discussions that arose at the time.

This project seeks to analyze issues related to health, access to armed conflict, and peace. These focus groups are linked to the “War and Peace” project by the Universidad de los Andes and the University of York. The project focused mainly on issues related to physical and mental health, the impact of the armed conflict on the community and the people who attended the focus groups, and access to health before and after the peace process with the FARC-EP [24]. The issue of disability arises from different testimonies from the participating population in the municipalities studied. Although not the main topic of the discussion, this emerging topic had a place in various moments of the sessions.

Issues related to disability are mainly associated with armed conflict, discrimination, and poor access to health, especially by the population with disabilities and their families, together with the impact of the conflict on this population or as the cause of the disability situation itself. These testimonies were taken from the recordings and verbatim transcripts of the sessions in each focus group.

### Data analysis

The data transcripts were analyzed using the Atlas Ti software. A set of codes was established to analyze the transcripts of the focus groups once they had been read for the first time. These codes are related to issues of disability and social, economic, political, and social inclusion, along with codes related to discrimination processes such as psychological and physical violence, poor access to health, abandonment, and mistreatment, among others.

The regulations were organized into categories and meta-categories. Possible codes and emerging categories were considered for the analysis of the transcripts. Once the interviews were coded, the results were collected by taking textual citations in the different categories of analysis to create a story that accounts for the situation of the population with disabilities in the department of Meta.

### Ethical considerations

The research was performed per the Declaration of Helsinki, and The Ethics Review Committee of Universidad de los Andes approved the study. Informed consent was obtained from each participant, and the researchers committed to safeguarding the confidentiality and anonymity of the data collected. Participants were informed of their right to refuse or withdraw from the study at any time without adverse consequences.

This paper is part of the “War and Peace” project by the Universidad de los Andes and the University of York [24]. As part of the project, all transcripts were made by a specialized third party who agreed to and signed confidentiality agreements to ensure anonymity and reduce biased interpretations. All project researchers had access to these transcripts once they were anonymized. Researchers conducting the fieldwork received additional mentoring on qualitative methodology, including ethical considerations. They received voluntary psychological support after the fieldwork to account for the personal challenges of conducting this research [24].

## Results

### Barriers in access to health for the population with disabilities in the department of Meta

The codification of the testimonies obtained in the focus groups showed that the population of Meta associates disability with different categories, such as vulnerability, violence, discrimination, access to health, care, and social protection, along with a close relationship with the armed conflict. Additionally, the most recurrent themes were related to the impact of the armed conflict on the participants, their mental and physical health, experiences associated with using health and medical services, changes over the years, and situations related to attention for the victim population. The following results show the barriers the victim population with disabilities has encountered in the department of Meta when trying to access health services. These barriers are caused by situations related to discrimination, conditions of vulnerability, obstacles caused by discrimination, or difficulties in accessibility in the provision of services and social protection, as well as barriers generated by violence.

### Vulnerability and discrimination against the victim population with disabilities

One of the main results presented in this research is the critical situation in which the victim population and the population with disabilities find themselves in the department of Meta. This occurs due to the precarious living conditions and the high levels of vulnerability in which this population finds itself as it is affected by armed conflict and acts of violence.

The situations caused by violence to which the population that participated in the focus groups has been exposed show that experiencing disability or being a relative of people with disabilities hinders the processes of recognition and reparation for the victim population. Furthermore, the costs generated by the experience of disability are not subsidized by the National Government or the Local Government.

The people who participated in the focus groups consider that the population with disabilities in Meta is vulnerable and lacking protection. For this population, which has seen its life affected by physical and psychological violence, forced displacement, and murder of relatives, living the experience of disability makes their situation, according to their testimonies, more challenging to cope with.

Woman 1: My son received a death threat. My son disappeared from Colombia and then appeared in Paraguay; I don’t know how. After six months, I heard about him, and in 2015 he was murdered in Paraguay. In 2015 I had my 22-year-old boy with cancer lying on a bed and my oldest son in a mental clinic; my situation was critical and very tough; I had an appointment on August 25, 2016, in the Victims Unit.

The victim population has shown that the educational, cultural, and social development of their children with disabilities depends exclusively on them and on their decision to move forward in the face of adversity and contexts of violence. They also recognize their difficulties paying for utilities (water, electricity, gas) and debts they have accrued over the years.

Woman 2: I want to save my son, and I don’t know how; I live with my mentally ill child, disabled, without a job, without opportunity; I owe rent, I owe two months, I owe the [utilities] bills, and I don’t have a job, I don’t know what to do.

Finally, this same woman recounts how her experience with the armed conflict led to the disability that her son is currently experiencing; a situation that, according to her, does not have sufficient support from health or social protection institutions, an asset that, if available, could lead to a psychological and health support, along with the financial backing to cover the extra costs of disability.

Woman 2: I think that my sick son needs me, but I’m sick, look: I have a headache that has not gone away with anything; I have been in this situation for more than two months, I am desperate and knowing that my spine is bad because they damaged my spine during the kidnapping, the second time they raped me the two who kidnapped me in the pregnancy of my child, I was seven months pregnant, because they were going to charge me for what my son’s father had not done, which was to pay them the extortion, so I had to pay them, and they charged me dearly, that’s why my child was born with a bad heart.

On the other hand, an issue raised by people with disabilities and their families in the focus groups was the different situations of discrimination that people with disabilities have faced in the department of Meta. Problems related to discrimination against the population with disabilities are related to labor and educational inclusion. Additionally, it has been shown that people with disabilities and of legal age usually have greater difficulty finding a job. These actions of discrimination and exclusion occur due to the disability experienced by this population.

Woman 3: She has a mental disability because of everything that is happening with her children, and she also needs financial support because she is not well. She can’t work because she can’t get a job; look how old she is; nobody gives her a job.

Finally, one of the main problems evidenced in the focus groups was the difficulty of the families of children with disabilities to access and participate in educational systems. According to them, the participation of children with disabilities in the classroom also depends exclusively on their parents or caregivers. This situation makes it difficult for families to work several hours or attend to other household issues.

Woman 4: I am a victim of the armed conflict, a displaced person, a mother of two children with disabilities because I cannot work a lot because my children need me because if he goes to study, I must be with him; I am a mother (companion) of the school.

The precarious situation of the families of people with disabilities and the lack of support provided by the government or national institutions to cover the extra expenses generated by the disability are recurring themes in the different sessions of the focus groups. In addition, it is highlighted that the victim population with disabilities and their families are in a situation of vulnerability and exclusion.

### Attention and social protection for the population with disabilities

When investigating the possible barriers to the population’s access to the health system, it is shown that one population with the most significant difficulties in accessing health services is the population with disabilities. In addition, it is shown that this population has problems accessing medicines and accessible transportation that allows them to get to controls and medical appointments. Furthermore, there is often not the necessary equipment to perform specific procedures.

Moderator: Perfect. So now, what you have seen right now is that health, first, is a right, but it is violated, and it is violated by all those things that you are saying: that there are no medicines, that there is no equipment, that there is no staff, that transportation is complicated, and there are some populations, as Sinovia said, that are more affected, such as the elderly, women ….

Woman 5: and persons with a disability as well.

One of the main problems regarding the access of people with disabilities to health services in the department of Meta is the lack of qualified medical personnel. In the focus groups, situations were narrated in which people with disabilities could not be cared for after arriving at the medical centers. This happens despite the long journeys that must be made to reach hospitals, emphasizing that, according to testimonies, this situation also occurs with older adults.

Woman 6: It is the same as the girl said; when you get sick, there is no care, especially for the elderly. Sometimes ill people come from the villages to the doctor, saying, “no, there are no doctors; there is no care for you.“

Another situation evidenced in the focus groups was the lack of differentiated care for people with disabilities. Considering that the health services do not have sufficient medical personnel, people with disabilities who participated in the focus groups stated that there is no support to travel to medical centers or support for caregivers of people with disabilities.

Woman 7: But here in the municipality of Vistahermosa, the health system ‘infringes our rights because, first, there are no doctors or care. When you try to get a medical appointment, you must wait a month or two for a medical appointment. And apart from that, if there is a transfer of a person with a disability, the accompanying person does not have transportation support.

Finally, it was shown that in the department of Meta, there are problems related to the prioritization of the population with disabilities within medical care processes, as well as difficulties with the certification processes of the people with disabilities, which has generated double exclusion practices and ableism.

Woman 8: Well, now, when it comes to the issue of why the elderly, pregnant women, and people with disabilities don’t get priority? Then one goes, and there is no priority; we are very unfair about that.

Woman 9: I carry a certificate that I have not thrown away because the day they tell me, I will show it. The certificate that a doctor gave me says: “handicapped, disabled person”. She said that at 38, she was in a wheelchair, and at 40, she couldn’t even use her arms; she was invalid, she had a degenerative spine, and she was already damaged up to this point.

Woman 10: When I was doing the papers for my brother’s death compensation, we realized that I was not registered as displaced, nor were my children with disabilities or anything; what did I have to do? Make an appointment, another appointment because apart from the fact that they are disabled, now a meeting of 10 or 12 doctors has to be made for them to give us an about what the disability is that each one has, and until that meeting was carried out the insurance could not confirm that I was indeed displaced with children with disabilities.

Thus, it can be evidenced that people with disabilities encounter different barriers to accessing health and social protection provided by the government due to practices of exclusion and discrimination. As stated in the testimonies, these situations have made it difficult for the population to be included at the social level in the department.

### Violence and its relationship with disability

Another of the results presented in this research relates to the relationship between disability and armed conflict, which is one of the leading causes of the disabling experience. As will be shown later, the victim population in the department of Meta, which either experiences the disability or is a relative of people with disabilities, associates, through testimonies reflected in the focus groups, the disability with some violent act.

One of the main results that arise from the atrocious relationship between disability and armed conflict is related to the lack of protection experienced by victims with disabilities in the department of Meta. This can be evidenced by the lack of guarantees at the time of being included in the State’s social services and by the low social protection that people with disabilities who have been victims of the armed conflict receive. An example of this situation is evidenced in the compensation by the national government to people with disabilities who have been victims of the armed conflict.

Woman 11: So, we were very affected; my children were very affected; I have a son who is deaf because of the bombs.

Moderator: And were you compensated for the disability?

Woman 11: No, this is how we are.

The problems related to compensation processes not only economically affect the victim population with disabilities but also negatively influence recognition functions as people with disabilities and victims of the Colombian armed conflict. In addition to the vulnerability in which this population lives, not being part of the reparation and recognition processes, as indicated by the people participating in the focus groups, generates emotional and psychological effects.

Woman 12: I stayed in “Justice and Peace” (program), but the psychological damage and personal injuries caused by my disability me were not recognized by the Victims Unit. I have fought for my wounds to be included because the law says so.

For the population, there is a direct relationship between war and disease, which, according to them, has brought an overload to the health system and suffering for the population and the families of the victims. On the other hand, it is shown that the people participating in the focus groups recognize and denounce the damage the armed conflict has caused. These affectations are related to physical injuries and psychological affectations, leading to disability.

Woman 13: The war has left many people with depression, insane people, and women with supremely high depression because a woman was impaled and raped. On top of that, they killed her children. I, at least, in my family, have one person who has gone through that; it makes the whole family sick because one suffers when one looks at a relative and cannot do anything. We are all displaced.

Faced with the question asked by the moderator, the people of the department of Meta state that the violence and the armed conflict have brought more disease and suffering to their population. The related affectations are mainly on a psychological level. The population recognizes the impact that the armed conflict has had on the general population, but above all, the effects that the conflict has had and its relationship with people with disabilities. Furthermore, it is evidenced that the population of the department of Meta relates the victim population with “sick” people or mental health-related disorders.

Woman 14: You were asking now if we believe that war brings more disease; of course, it did. How many victims are there in Colombia? At least in the department of Meta, there are almost two million seven hundred thousand victims, at least two million households, and two or three people per household. There are six million sick people because we are all ill.

Woman 15: Even more, because the war brought us psychological damage.

The relationship between armed conflict, health, and disability is one of the most discussed topics during the focus groups. In these, the experiences and feelings that the armed conflict has left in the victim population, people with disabilities, and their families became known. They all agree that the conflict and acts of violence have triggered the effects on the physical and psychological health of the people in this department.

## Discussion

This research analyzed how people with disabilities, the victim population with disabilities, their caregivers, and their families find barriers to access to health services in the department of Meta, Colombia. The results show that this population experiences barriers to access due to the high levels of vulnerability and lack of protection in which they find themselves. In addition, this population has experienced high levels of discrimination by the Colombian government, the entities that provide health services, and the people around them. Finally, the population with disabilities states that the armed conflict between the government and the guerrilla groups mainly caused physical and psychosocial limitations. The information was obtained from focus groups in the department of Meta.

As evidenced in other research [25–28], the population with disabilities in Colombia faces more significant difficulties in accessing services such as education, health, and formal employment. In addition, it has been evidenced that the victim population encounters different barriers to accessing the same benefits [29–31]. The victim population with disabilities is in a situation of double or triple exclusion when they want to access these services, considering the high levels of inequality, vulnerability, and poverty they face [19].

This study has shown that the victim population with disabilities faces barriers when trying to access health services due to long distances to reach care centers, treatment costs, poor care, poor health practices, lack of trust in physicians, and the lack of medical services when required [16]. Additionally, it exposes the socioeconomic vulnerability of this population in the department of Meta due to the lack of attention from the government and the low levels of social protection.

Furthermore, the victim population with disabilities and their families experience high levels of unemployment and informality [11], which is related to low levels of schooling, the increase in debts due to late payments for utilities (water, gas, and electricity) and difficulties in obtaining or renting a home.

This research also manifests how the population with disabilities directly relates to the armed conflict with disability and disease. This relationship shows a disapproving perception of the population with disabilities, which can increase the levels of discrimination and exclusion in this population [32]. Most people in this research assure that their limitations and illnesses result from violent acts and armed conflict.

Another vital relationship derived from this research is the high participation of women in the focus groups compared to men’s involvement. Women’s participation may be related to the fact that, in the Colombian armed conflict, they have been more affected by armed displacement, sexual violence, gender-based violence, barriers to accessing formal employment [33], [34], which makes them more likely to debate, discuss their current situation and advocate for themselves.

The population also expressed in the focus groups the difficulties children with disabilities and their families encounter when trying to gain access to educational institutions and centers. As shown in this research, this population’s access to academic centers is subject to the attendance of relatives at said educational centers, which evidences the need for more reasonable adjustments and differentiated actions to guarantee the educational inclusion of this population. In addition, this situation makes it difficult for families of people with disabilities to access formal jobs or participate in social and daily activities in the department of Meta.

Finally, this study shows the country’s difficulty in the certification of people with disabilities and the information systems for the characterization and location of this population. Because of this, victims with disabilities have encountered barriers to access the reparation and rehabilitation processes provided by the national government [35]. This has prevented this population and their families from accessing services for the victim population [35].

In Colombia, the Victims Law 1448 [23] and the Statutory Disability Law 1618 [36] protect the victim population with disabilities and strive for this population to be included in physical, psychological, and psychosocial rehabilitation processes. Moreover, they recognize the importance of including this population in the state’s different services, the health service. However, these laws, which have solid content and a disability perspective based on the Convention on the Rights of Persons with Disabilities [1], have not been fully implemented in most Colombian territories [37–40]. This situation affects the inclusion processes and increases the problem of vulnerability, exclusion, and violence to which the population in the country is exposed.

## Conclusions

Many problems affect the population with disabilities and the victim population in Colombia today. The armed conflict has left millions of victims, thousands disappeared, and approximately seven million people have been displaced due to violence [41]. The consequences of the conflict have made it impossible for millions of people with disabilities and victims to access the health systems in the country.

This study shows how the Colombian government, in the post-accord, has yet to establish adequate policies to eliminate or even reduce the barriers that make it difficult for this population to access services such as health, education, housing, and social protection. Furthermore, there are no policies that prevent re-victimization when accessing health services. Additionally, the government’s inability to provide security levels in the country’s most remote areas is evidenced. Further, in these areas, events still violate the rights of victims with disabilities.

This population requires the national and local governments to implement laws that protect them from violations of the human rights of people with disabilities and the victim population. An urgent call is made for the international community to pressure the Colombian government to find different ways to include the population with disabilities in the social services provided by the State.

The Colombian population with disabilities and the victim population of the country’s armed conflict deserve to be recognized, not only for what is enshrined in national and international laws but also for the immense historical debt the government has to this population. A population that, to this day, only finds shelter and a helping hand in its community.

## Electronic supplementary material

Below is the link to the electronic supplementary material.

Supplementary Material 1

## Data Availability

The datasets generated during the current study are not publicly available because they contain information that could compromise research participant privacy/consent but are available from the corresponding author upon reasonable request.
