# Supplementary material for: Access to health services during the Colombian armed conflict: a challenge for the population with disabilities in the department of Meta
Source: BMC Health Serv Res. 2023 Jun 13;23:628. doi: 10.1186/s12913-023-09472-x (PMC10265762; doi:10.1186/s12913-023-09472-x)
Supplement: Supplementary file 1 — Supplementary Material 1 [file 12913_2023_9472_MOESM1_ESM.docx]

**FIELDWORK GUIDE**

**Focus group**

**Focus group guiding questions**

**Group time:** 1 hour approximately

**Number of focus groups per municipality**: three groups: one group of men, one group of women, and one mixed group.

1. What is health for you?
2. What is illness?
3. How do you perceive your state of health?
4. How do you rate the health of the people living in your municipality?
5. What are the most frequent health problems (be aware of differences according to gender)?
6. Where do they go or what do they do when they get sick?
7. Who is the caregiver in your home?

*Conflict*

1. Do you think the armed conflict has affected the provision of health services?
2. What changes do you perceive before and after the signing of the Peace Agreement?
3. In relation to your health, what were your concerns 10 years ago? What are your concerns now?
4. What did they use to do in conflict situations when they got sick or when they had any health-related problem?
5. Do you think there are any health problems related to the history of conflict in the municipality? Which ones?
